# Photochemistry and Radical Chemistry under Low Intensity Visible Light Sources: Application to Photopolymerization Reactions

**DOI:** 10.3390/molecules190915026

**Published:** 2014-09-18

**Authors:** Jacques Lalevée, Fabrice Morlet-Savary, Céline Dietlin, Bernadette Graff, Jean-Pierre Fouassier

**Affiliations:** 1 Institut de Science des Matériaux de Mulhouse (UMR CNRS-UHA 7361), 15 rue Jean Starcky, 68057 Mulhouse Cedex, France; 2 ENSCMu-UHA, 3 rue Alfred Werner, 68200 Mulhouse, France

**Keywords:** radical photopolymerization, free radical promoted cationic polymerization, photoinitiators, organometallic photocatalysts, organophotocatalysts, soft conditions, photoredox catalysis

## Abstract

The search for radical initiators able to work under soft conditions is a great challenge, driven by the fact that the use of safe and cheap light sources is very attractive. In the present paper, a review of some recently reported photoinitiating systems for polymerization under soft conditions is provided. Different approaches based on multi-component systems (e.g., photoredox catalysis) or light harvesting photoinitiators are described and discussed. The chemical mechanisms associated with the production of free radicals usable as initiating species or mediators of cations are reported.

## 1. Introduction

In photochemical reactions, both the absorbing compound (AC) and the kind of light source used are decisive [1,2,3,4,5,6,7,8,9,10,11,12,13,14]. Indeed, the obtention of a given product P or the formation of reactive intermediates RI is a function of: (i) the amount of light absorbed I_abs_, that depends on the characteristics of the irradiation source (wavelength λ_S_ and intensity I_0_) and the AC absorption properties (wavelength λ_AC_ and molar extinction coefficients ε) and (ii) the quantum yield Φ of the reaction that is connected with the excited state processes involved. It is obvious that other parameters being kept unchanged, the amount of P or RI increases with I_0_. In the same way, in the case of a poorly reactive ACs (low Φ), the P/RI amount can remain acceptable provided a high I_0_ is available. An interesting topic refers to the case where one has to use visible light (e.g., to avoid hazardous UV light) and low intensity sources (e.g., available cheaper and safer devices, imposed requirements): under these experimental conditions referred to as soft irradiation conditions, the challenge is how to find very reactive ACs to get a suitable P/RI production. This still represents a challenge in preparative photochemistry and organic synthesis [1,2,3,4,5,6,7,8,9,10,11,12,13,14]. For example, a lot of works are currently devoted to this problem in photoredox catalysis that is often carried out with organometallic complexes or metal-free organic compounds as ACs under low intensity visible light emitting sources [15,16,17,18,19,20,21,22,23,24,25,26,27,28]. A problem that has to be carefully considered relates to the detrimental presence of oxygen in any process that involves a long lived AC triplet state T_1_ (oxygen is a strong quencher of T_1_; the rate constant for quenching is often close to the diffusion limit) or/and the occurrence of a radical species (the addition to oxygen easily leading to a peroxyl radical) [2]. 

In the present paper, we wish to discuss the use of soft irradiation conditions in the field of photopolymerization reactions. Usually (and often industrially), such reactions are conducted under high intensity UV or near UV lights [2,3,4,5,6,7,8,9,10,11,12,13,14] e.g., in the radiation curing field to get very high cure speeds for coating production. In various sectors (imaging, graphic arts, optics, microelectronics, stereolithography, medicine, dentistry, nanotechnology…), soft irradiation conditions under air may be required. Although huge efforts have already been done to develop efficient photoinitiating systems PIS [2], this additional constraint calls for the development of new very reactive ACs and novel strategies for the design of PISs able to start the polymerization. In the following, we will detail this approach.

## 2. Results and Discussion

### 2.1. The Novel Strategy

In the photopolymerization area, the main reactions encountered are free radical polymerization (FRP), cationic polymerization CP and free radical promoted cationic polymerization (FRPCP) of monomers or/and oligomers. They are photoinduced,* i.e.*, a photoinitiating system (PIS) is necessary. Usual and known PISs contain: (i) a cleavable photoinitiator PI (Type I PI); or (ii) a PI and a co-initiator (electron/hydrogen donor) or a photosensitizer PS and a PI (Type II PI); or (iii) several components (e.g., PS, PI, additive). 

The rate of a polymerization Rp is a function of I_abs_ and φ_i_ (the initiation quantum yield that corresponds to the number of starting chains per photon absorbed), φ_i_ being a function of the relative efficiency of the processes involved in the generation of the initiating species IS [2]. The amount of ISs and, in turn, the Rp value, are dependent on I_0_. 

In FRP and FRPCP where radicals are present, the oxygen inhibition effect can be counterbalanced when using:

(i) high intensity light sources (e.g., a few W/cm² with a Hg lamp or focused laser beams) as the high amount of IS easily counterbalances the loss due to the oxygen quenching reactions;
(ii) highly viscous media (viscosities > 1000 cp) where the diffusion rate constant k_diff_ and accordingly all bimolecular rate constants level off: this means that the oxygen quenching of radicals is therefore slowed down;
(iii) thick samples; in thin samples, a very fast re-oxygenation is observed in the course of the photopolymerization, leading to a higher oxygen inhibition.

When using soft experimental conditions (under exposure to e.g., household halogen, fluorescent bulbs and LED lamps, sun; intensity 2–10 mW/cm^2^) under air and, moreover, in low viscosity media (80–100 cp), the situation is obviously completely different. None of the conventional PISs developed so far can work in these conditions. In recent years, we have designed novel PISs (based on three-component combinations‒TCC) that are able to meet this challenge and probably open new opportunities. Such TCCs consist of one AC and two additives A and B. The conditions that have to be fulfilled are: (i) excellent light absorption properties for the AC compound; (ii) preference for a route that involves a short lived first excited singlet state S_1_ which avoids any substantial oxygen quenching; (iii) occurrence of strong and selective interactions between the three components; (iv) avoiding oxygen inhibition in the medium as much as possible; (v) production of efficient radical or/and cationic IS. According to what was available in the literature and in our previous works, our starting idea (see e.g., in [29,30,31,32,33,34,35,36,37,38,39,40,41,42,43]) was to combine a silane R_3_SiH and an iodonium salt with a new series of suitable and carefully designed ACs with absorption maxima tunable over the 385–700 nm wavelength range and together with dramatically improved absorption properties. Silanes are well known for generating silyl radicals R_3_Si^•^ [29] and undergoing reactions with oxygen. Iodonium salts Ph_2_I^+^ are easily reducible in electron transfer reactions with: (i) the S_1_ state of many ACs [2,3,4,5,6,7,8,9,10,11,12,13,14] (^1^AC/Ph_2_I^+^ interactions with almost diffusion controlled rate constants are observed; two drawbacks are, however, a possible back electron transfer or a too short lifetime, due to competitive deactivation pathways, which decreases the electron transfer quantum yield) and (ii) radicals (e.g., 2.6·10^6^ M^−1^·s^−1^ for the R^•^/Ph_2_I^+^ interaction rate constant where R^•^ stands for the tris(trimethylsilyl)silyl radical [29,30,31,32,33,34,35,36,37,38,39,40,41]). 

**Scheme 1:**
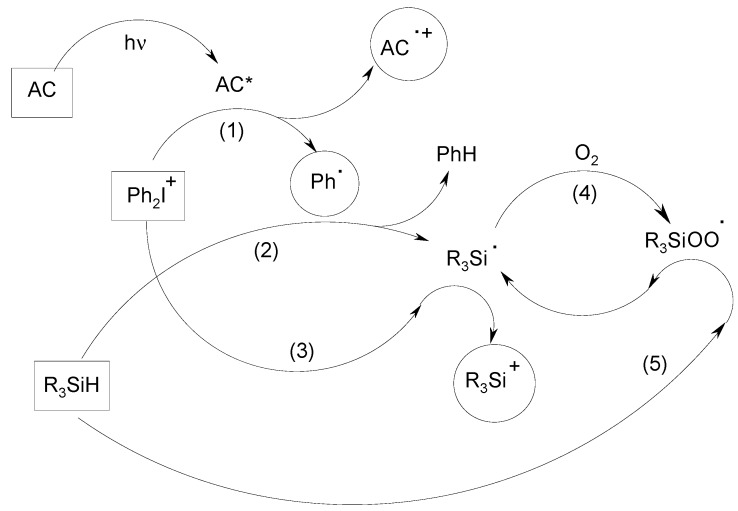
Chemical mechanisms involved in the AC/Ph_2_I^+^/R_3_SiH systems.

Scheme 1 summarizes the overall mechanism encountered in such an AC/Ph_2_I^+^/R_3_SiH TCC. The most efficient interaction concerns AC/Ph_2_I^+^ (1) which leads to a dissociative Ph_2_I^•^ radical and thus to Ph^•^ + PhI. The formed phenyl radical reacts with the silane: a silyl is generated (2). The R_3_Si^•^/Ph_2_I^+^ interaction produces a silylium cation R_3_Si^+^(3). The silyl consumes oxygen (4) and the formed peroxide is converted into a new silyl species (5). Moreover, phenyls and silyls efficiently add to acrylate double bonds (1.9·10^8^ and 0.22·10^8^ M^−1^·s^−1^ on methylacrylate, respectively [29]) and a silylium cation is a very reactive species for the ring opening of an epoxide (the silylium/cyclohexeneoxide interaction energy is highly exothermic: −152 kJ/mol [29]; this value is noticeably more favourable than that for a carbocation). On the basis of these data, one can expect a high reactivity (high φ_i_) for the TCC that can counterbalance a low I_0_ and I_abs_: this should be decisive for an application under soft irradiation conditions. Therefore, this TCC is a worthwhile and versatile dual system as it allows the formation of radicals (Ph^•^ and R_3_Si^•^), cations (R_3_Si^+^) and radical cations (AC^•+^): AC governs the absorption properties and (to a lesser extent) the reactivity but the nature of radicals and cations formed is not dependent on AC. FRP is initiated by Ph^•^ and R_3_Si^•^. The photoinitiation through AC^•+^ and R_3_Si^+^ leads to CP and FRPCP, respectively. 

**Scheme 2:**
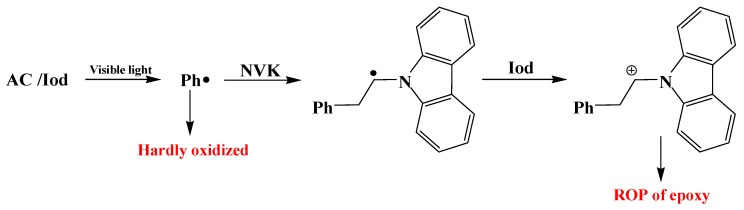
Role of NVK in AC/Ph_2_I^+^(or Iod)/NVK systems.

*N*-vinylcarbazole (NVK) can play the same role as the silane (Scheme 2). The phenyl adds to the NVK double bond and forms an easily oxidizable carbon centred radical on NVK (Ph-NVK^•^) [31]. 

**Scheme 3:**
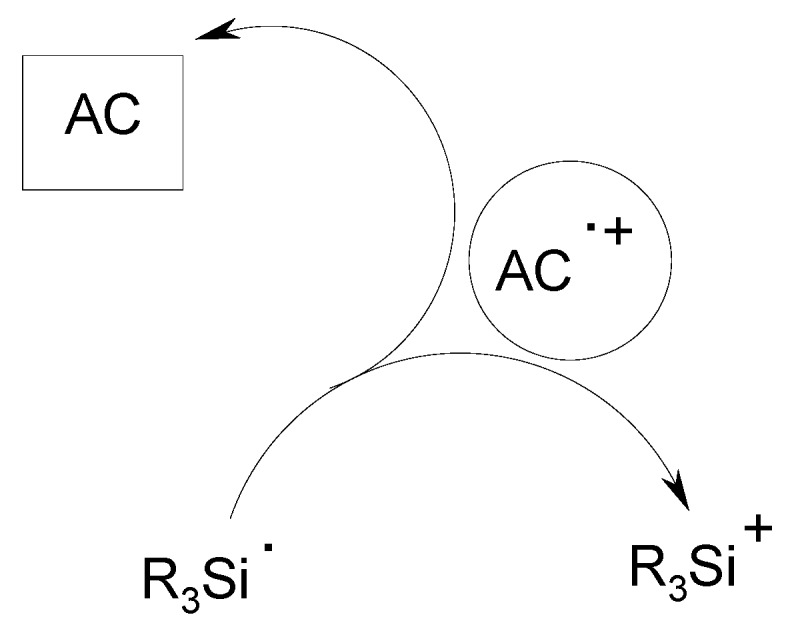
Oxidation of silyl radicals by AC^•^^+^.

When using organometallic complexes as AC, the AC^•^^+^ radical cation is reduced by R_3_Si^•^ or Ph-NVK^•^. This is an additional way to produce a silylium or a Ph-NVK^+^ cation [31]. The organometallic complex is regenerated leading to a photocatalyst behaviour (Scheme 3): the photoinitiator becomes a photoinitiator catalyst PIC. The photoredox system follows an oxidation cycle.

**Scheme 4:**
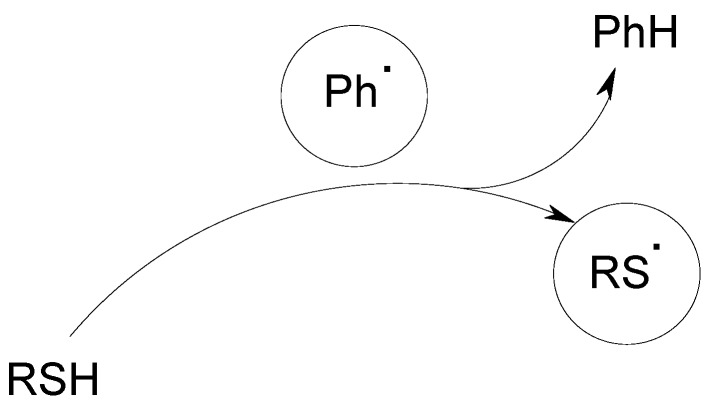
Formation of thiyl radicals in the AC/Ph_2_I^+^/RS-H systems.

The change of the silane for a germane or a borane was also checked and the same general mechanism occurs [2,31]. A thiol can also be used instead of a silane. In that case, a thiyl radical is formed (Scheme 4).

**Scheme 5:**
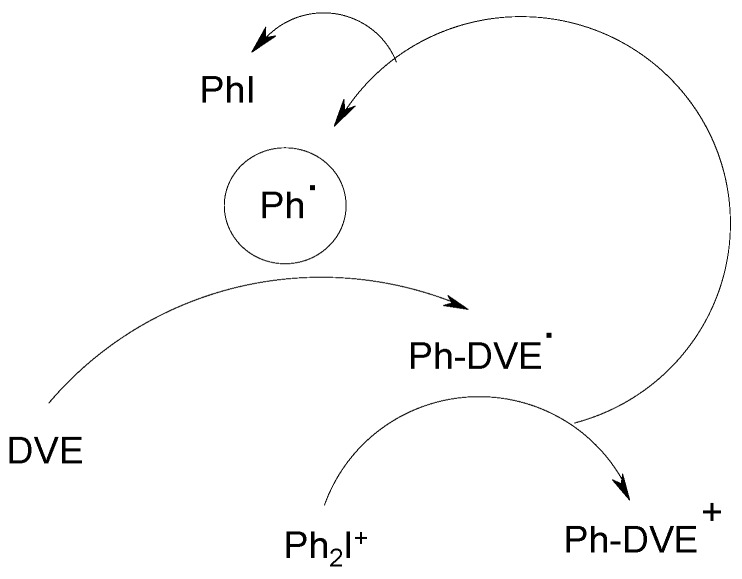
Initiating species formed in presence of a vinylether monomer (DVE is a divinylether; see [42]).

When using a vinyl ether monomer instead of an epoxide, the cationic polymerization does not occur in the presence of radical scavengers (e.g., oxygen, phenyl-*N*-*t*-butylnitrone). This suggests a FRPCP process where the cation centered on the vinyl ether unit (Ph-VE^+^) formed in Scheme 5 is the initiating structure [42].

**Scheme 6:**
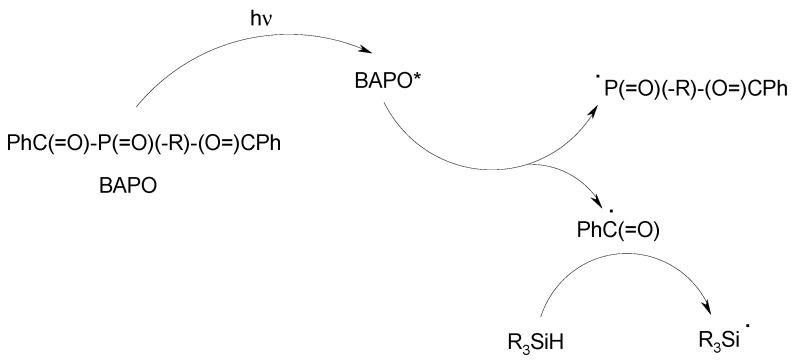
The BAPO/R_3_SiH photoinitiating system.

Although this is somewhat different, a two-component system based on a cleavable photoinitiator (Type I PI, e.g., a bisacylphosphineoxide derivative‒BAPO) and a silane exhibits a polymerization ability higher than that of the Type I PI alone (Scheme 6). 

Indeed, BAPO generates an efficient phosphorus centred initiating radical (radical addition rate constant to acrylate k_add_ ~ 10^7^ M^−1^·s^−1^) [2,43] and a relatively low efficient benzoyl counter radical (k_add_ ~ 10^5^ M^−1^·s^−1^): in BAPO/silane, this latter radical is replaced by an interesting (see above) silyl radical. 

**Scheme 7:**
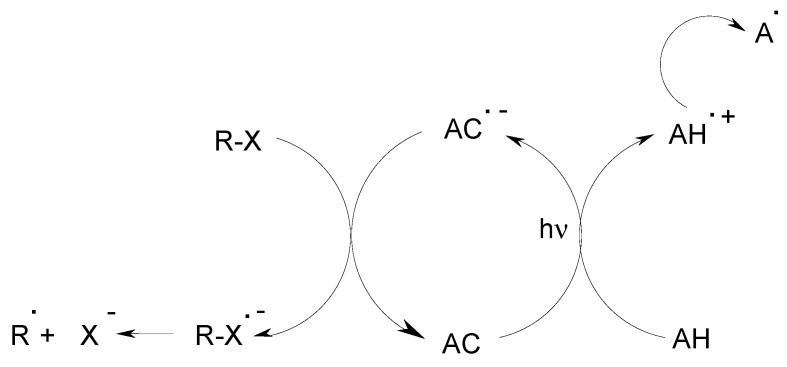
Reduction catalytic cycle in AC/AH/RX photoinitiating systems.

The newly developed ACs in combination with an amine AH and an alkyl halide RX can also be successfully used in photoredox catalytic reduction cycles as metal-free PICs (Scheme 7). This is in contrast with other classical dyes (Eosin, Rose Bengal…) mentioned in organic synthesis [27,28,31], that cannot allow the design of AC/AH/RX efficient three-component PISs of polymerization.

All these TCCs should ensure the photoinitiation of the FRP of acrylates, the CP and the FRPCP of epoxides and vinylethers as well as the polymerization of acrylate/epoxide AEP (or vinyl ether) blends for getting interpenetrating polymer networks IPN. Thiol-acrylate polymerization TAP and thiol-vinyl ether polymerization TVEP (using the thiyl as an initiating radical) should also be feasible. 

### 2.2. Performance of the Novel Photoinitiating Systems

We will show here some examples that illustrate both the high photochemical/chemical reactivity and polymerization ability of the new proposed TCCs under various soft irradiation devices: low intensity Xe or Xe-Hg lamps (<40 mW·cm^−2^ in the 370–800 nm range), laser diodes (monochromatic lights; output 10–100 mW/cm^2^; operating at selected wavelengths such as 405, 457, 473, 532, 635, 780 nm …), LEDs (quasi monochromatic light; band pass ~ 40 nm; output 7–20 mW/cm^2^ at a selected wavelengths: 395, 405, 455, 470 nm* etc.* …. ), household halogen lamps (~12 mW·cm^−2^ in the 370–800 nm range) and LED or fluorescent bulbs (<15 mW/cm^2^ in the 380–800 nm range), sun (white light with near UV emission; <5 mW/cm^2^ in the 350–800 nm range). Some TCCs are efficient: the FRP of low viscosity acrylates is perfectly acceptable in laminates and can be good under air (see below); the FRP of methacrylates can also be done and is characterized by excellent conversion-time profiles. TAP and TVEP are favourably carried out in laminates. Excellent FRPCP and AEP under air are noted with almost all systems (except when using divinylethers) and a large range of excitation wavelengths can be used from the UV to the red. Examples of high performances initiating systems will be now given; some examples of TCCs will also be provided.

### 2.3. Development of Light Harvesting Photoinitiators 

The search for new Type I or Type II PIs is currently the subject of many research works (e.g., for examples, see in [44,45,46,47,48,49,50,51,52,53,54,55,56]) through: (i) the design of new structures and (ii) the modification of existing structures. As recalled above, the amount of light absorbed by the ACs and the photochemical/chemical reactivity of ACs constitute the key points for the design of high performance PI systems [2].

**Figure 1:**
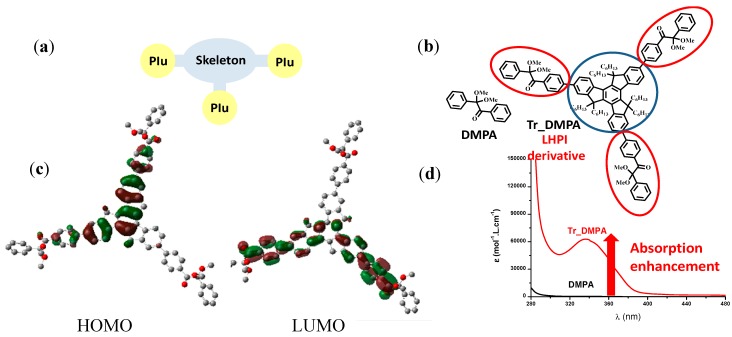
Comparison of the light absorption properties of a LHPI derivative (Tr-DMPA)* vs.* those of the corresponding DMPA moiety: (**a**) structure for a LHPI derivative; (**b**) chemical formula for DMPA and Tr-DMPA; (**c**) Molecular orbitals for Tr-DMPA and (**d**) UV-visible absorption spectra for DMPA and Tr-DMPA.

For example, in Type I PIs, the initiating radicals are generated by a homolytic single bond cleavage upon light excitation e.g., in 2,2'-dimethoxy-2-phenylacetophenone (DMPA, Figure 1) or in BAPO, the C-C cleavage process being usually very fast (<1 ns). Such PIs are currently based on relatively small molecular structures (e.g., aromatic ketones) and absorb in the UV (e.g., DMPA) or near UV/visible (e.g., BAPO). Difunctional (DiPI) and macromolecular Type I PIs (MaPI) have been developed (see many references in [2,3,4,5,6,7,8,9,10,11,12,13,14]). However, their absorption properties are either: (i) not modified and obviously resemble those of the starting individual unit (due to the lack of interaction between these units) or (ii) only slightly changed as resulting from a small molecular orbital MO coupling.

**Scheme 8:**
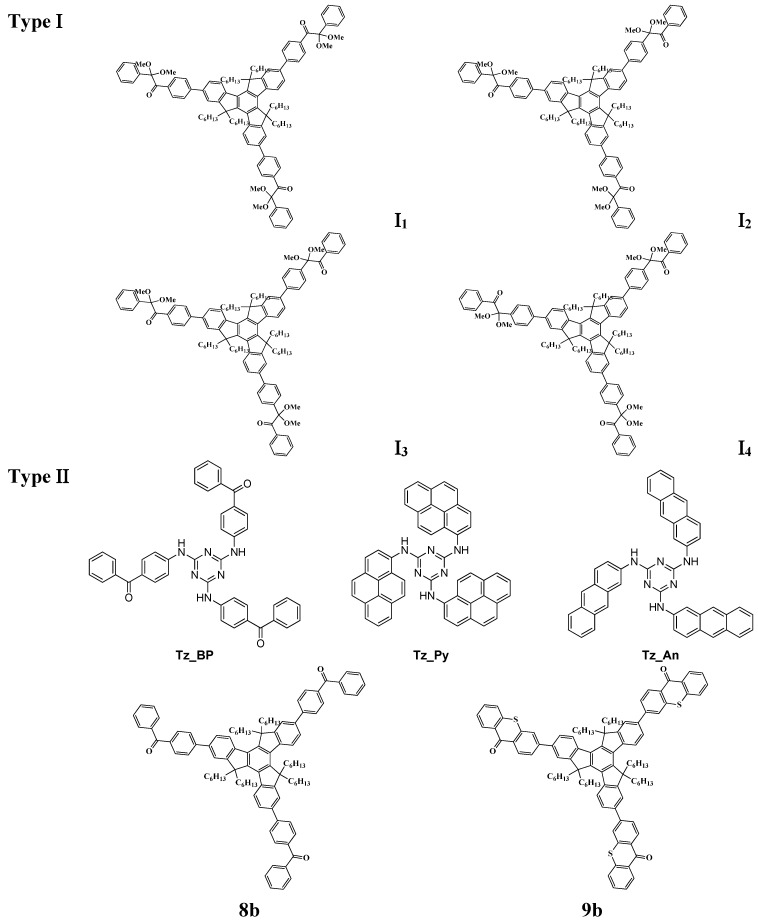
Examples of LHPIs as Type I and Type II photoinitiators.

**Figure 2:**
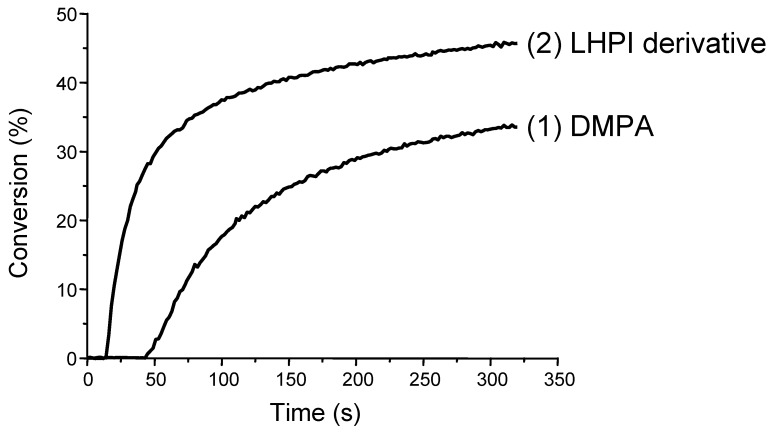
Photopolymerization profiles of trimethylolpropane triacrylate (TMPTA) in laminate upon a halogen lamp exposure in the presence of (**1**) DMPA or (**2**) the corresponding Tr-DMPA LHPI (from [57]).

Truly red-shifting the absorption and enhancing the light absorption intensity in PI architectures while keeping a high photochemical/chemical reactivity require a strong modification of the chemical structures. Everybody is aware that a strong MO coupling leads to dramatically improved absorption properties (red-shifted wavelengths λ_max_, higher molar extinction coefficients ε). Striking results in terms of λ_max_ and ε and excellent efficiencies in photopolymerization reactions have been recently obtained [57] using a suitable skeleton decorated with several cleavable units PIu where both a substantial electron delocalization and an important charge transfer never attained so far occur (Figure 1). Such PIs are characterized by unprecedented light absorption properties (ε > 100,000 M^−1^·cm^−1^) and referred as Light Harvesting Photoinitiators (LHPIs). Type II PIs based on a similar approach were also proposed (Scheme 8). They involve a PI (consisting e.g., in a core linked to electron acceptor aromatic ketone moieties) and an electron/hydrogen donor. Some examples of recently proposed Type I and Type II LHPIs are gathered in Scheme 8. In Figure 2, it can be observed that their performance as radical PIs is much better as revealed by the higher polymerization rates and final conversions.

### 2.4. Design of Systems Generating Silyl Radicals

**Figure 3:**
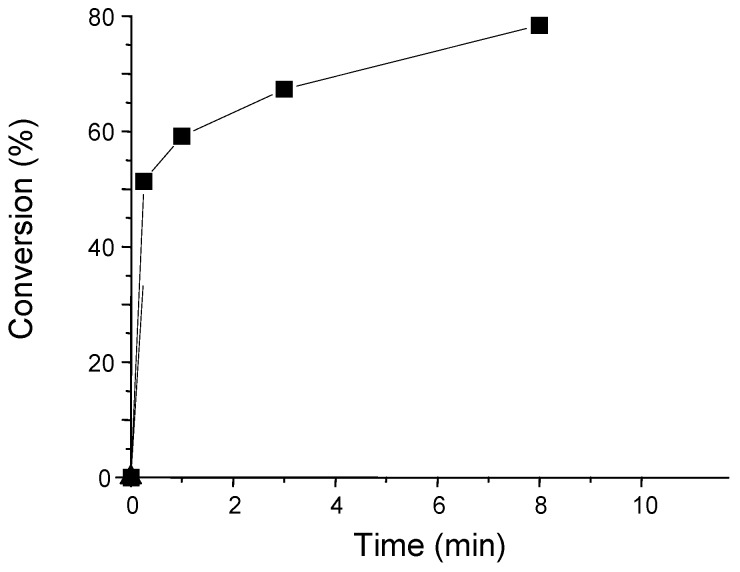
Photopolymerization profile of an epoxy acrylate formulation (Ebecryl 605) using the BAPO/(TMS)_3_SiH (2%/3%, w/w) photoinitiating system upon a sunlight exposure. Under air.

The silyl radicals are characterized by intrinsic advantages to initiate polymerization processes (see above). Accordingly, different silane containing PISs were specifically developed. As an example, the BAPO/tris(trimethylsilyl)silane couple is able to induce the radical polymerization of viscous resins upon sunlight exposure under air (Figure 3) [42].

### 2.5. Photoredox Catalysis in Photopolymerization Reactions Using Cheap and Non-Toxic Metal Complexes 

Photoredox catalysis has recently gain momentum in the synthetic community thanks to the breakthrough reported by different authors [15,16,17,18,19,20,21,22,23,24,25,26,27,28]. The outstanding reactivity of the excited state of a given ruthenium or iridium complex in the presence of an oxidative or reductive quencher under low intensity irradiation leads to new chemoselective bond activations. This strategy has opened new avenues for e.g., the formation of CC bonds, the reduction of organic halides, the atom transfer radical addition to unactivated terminal alkenes, or the conversion of alcohols to the corresponding halides. All these processes are characterized by an exceptional functional group tolerance, wide scope, very mild reactions conditions, and minimization of waste products. Recently (2010-to present), this approach has been extended to the polymer area through the development of new versatile photocatalysts as ACs and PIs (photoinitiator catalysts‒PIC) working under LED bulbs, sun, halogen lamp or green fluorescence bulbs as irradiation sources [31,34,35,36,37,38,39,40,41]. 

This approach paves the way for the design of a novel class of high performance PICs for both radical and cationic polymerizations and brings new properties (some of them are hardly accessible with the current systems), for example: (i) almost no PIC is consumed; (ii) the spectral photosensitivity extends from the UV to the visible range; (iii) the excitation under blue, green or red laser or LED lines is feasible; (iv) low light intensity light sources can be used; (v) the same initiating species are formed whatever the kind of PICs rendering the PISs highly tunable; (vi) the production of the initiating radicals for the FRP of acrylates or the cationic species in the CP/FRPCP of epoxides is quite easy and (vii) the polymerization reactions can be carried out under air. Expensive (and sometimes toxic) Ir or Ru complexes can be used as PICs: usual compounds and novel derivatives with suitable ligands have been tested [34,35,36,37,38,39,40,41]. The search for other metal centred PICs and metal-free PICs (based on so-called organophotocatalysts OPC) as well as oxidation and reduction agents remains fascinating. Two recent examples are selected here.

#### (a) Copper and iron complexes as cheap or lower toxicity photocatalysts

**Scheme 9:**
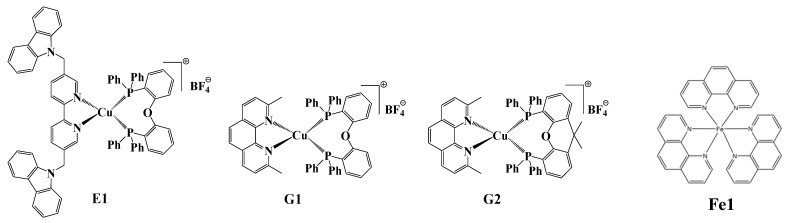
Recently proposed copper and iron complexes as PICs.

**Figure 4:**
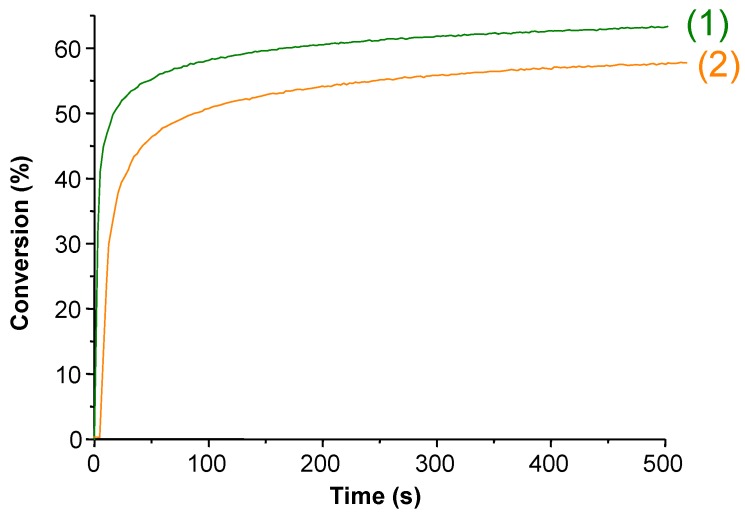
Photopolymerization profiles of TMPTA in the presence of G_1_/iodonium salt/NVK (0.2%/2%/3%, w/w/w) upon the LED at 405 nm (curve 1) or LED at 455 nm (curve 2) exposure in laminate.

Some examples of recently proposed copper complexes are given in Scheme 9 [58]. In combination with a iodonium salt and N-vinylcarbazole (NVK), some of these complexes (e.g., G1) act as efficient PICs for the FRPCP or FRP upon particularly soft irradiation conditions (e.g., halogen lamp). They work according to the chemical mechanisms already presented in Scheme 1 and Scheme 2. As shown in Figure 4, their efficiency under near UV and visible LEDs can be excellent. Thiol-acrylate, thiol-ene and AEP processes can also be promoted by light excitation of these systems.

#### (b) The photoredox catalysis using novel organophotocatalysts

**Scheme 10:**
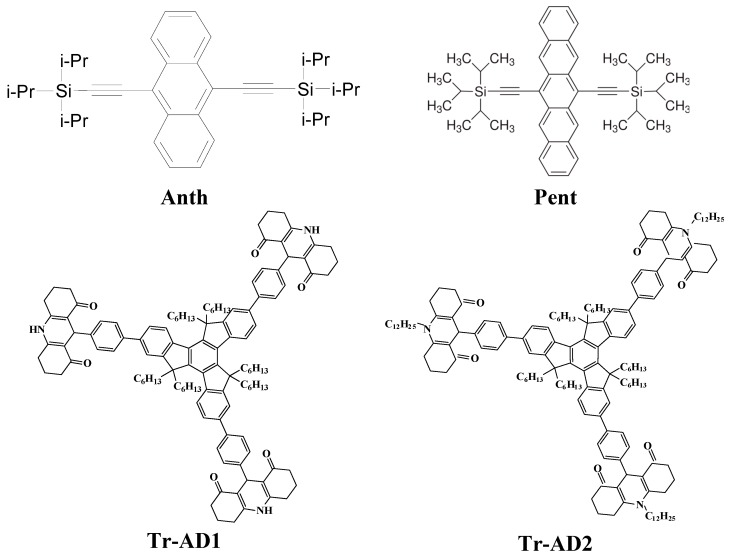
Examples of recently reported OPCs.

Some examples of organophotocatalysts OPCs reported in the last years are shown in Scheme 10 (see e.g., [31,42]). These OPC/iodonium salt/NVK or OPC/iodonium salt/silane combinations are very efficient to initiate FRPCP and FRP as well as thiol-acrylate, thiol-ene and AEP processes upon very soft irradiation conditions (e.g., halogen lamp, fluorescence bulbs, household LEDs …). Excitation up to 635 nm lights is also feasible. 

## 3. Experimental Section

The experimental details of the examples reported here can be found in the references [31,42,57,58].

### 3.1. Steady State Photolysis Experiment

The UV-vis spectra were recorded using a JASCO V-530 UV/Vis spectrophotometer.

### 3.2. Redox Potentials

The oxidation or reduction potentials (*E_ox_* or *E_red_ vs.* SCE) were measured in acetonitrile by cyclic voltammetry with tetrabutylammonium hexafluorophosphate (0.1 M) as a supporting electrolyte (Voltalab 6 Radiometer). The working electrode was a platinum disk and the reference electrode was a saturated calomel electrode (SCE). Ferrocene was used as a standard, and the potentials determined from the half peak potential were referred to the reversible formal potential of this compound (+0.44 V/SCE). The free energy change Δ*G* for an electron transfer between FS and iodonium salt can be calculated from the classical Rehm-Weller equation: Δ*G* = *E*_ox_ – *E*_red_ – *E*_S_ + *C*; where *E*_ox_, *E*_red_, *E*_S_, and *C* are the oxidation potential of FS, the reduction potential of iodonium salt, the excited state energy of FS, and the electrostatic interaction energy for the initially formed ion pair, generally considered as negligible in polar solvents. 

### 3.3. ESR Spin Trapping (ESR-ST) Experiment

ESR-ST experiment was carried out using an X-Band spectrometer (MS 400 Magnettech). The radicals were generated at room temperature upon the Xe–Hg lamp exposure under N_2_ and trapped by phenyl-*N*-*t*-butylnitrone (PBN) according to a procedure described elsewhere in detail. The ESR spectra simulation was carried out using the WINSIM software.

### 3.4. Photopolymerization Experiments

For photopolymerization experiments, the conditions are given in the figure captions. The photosensitive formulations were deposited on a BaF_2_ pellet under air or in laminate (25 µm thick) for irradiation with different light sources. The evolution of the epoxy group content of EPOX and the double bond content of TMPTA were continuously followed by real time FTIR spectroscopy (JASCO FTIR 4100) at about 790 cm^−1^ and 1630 cm^−1^, respectively.

## 4. Conclusions

The present paper has briefly summarized recent achievements in the field of radical and cationic photopolymerization, as well as thiol-ene or simultaneous radical/cationic reactions, under soft irradiation conditions. The design of new photoinitiators, photoinitiator catalysts and photoinitiating systems has provided an important step forward as polymerization reactions in low viscosity media under low intensity visible light (400–700 nm) and often in the presence of oxygen become now feasible. In these experimental conditions, most of them are better than reference PISs. This should probably open new opportunities in the applications of such reactions in traditional sectors and, undoubtedly, in innovative emerging areas. Some of the newly developed organometallic photocatalysts and organophotocatalysts might also be of interest e.g., in organic photochemistry or organic photoredox catalysis under soft irradiation conditions. The design, synthesis and testing of other PIs and PICs for FRP, CP and FRPCP are in progress.
